# Environmental biodegradability of recombinant structural protein

**DOI:** 10.1038/s41598-020-80114-6

**Published:** 2021-01-08

**Authors:** Yuya Tachibana, Sunita Darbe, Senri Hayashi, Alina Kudasheva, Haruna Misawa, Yuka Shibata, Ken-ichi Kasuya

**Affiliations:** 1 Division of Molecular Science, Faculty of Science and Technology, Gunma University, 1-5-1 Tenjin, Kiryu, Gunma 376-8515 Japan; 2 Gunma University Center for Food Science and Wellness, 4-2 Aramaki, Maebashi, Gunma 371-8510 Japan; 3 Spiber Inc., 234-1 Mizukami Kakuganji, Tsuruoka, Yamagata 997-0052 Japan

**Keywords:** Biopolymers, Biomaterials - proteins, Sustainability

## Abstract

Next generation polymers needs to be produced from renewable sources and to be converted into inorganic compounds in the natural environment at the end of life. Recombinant structural protein is a promising alternative to conventional engineering plastics due to its good thermal and mechanical properties, its production from biomass, and its potential for biodegradability. Herein, we measured the thermal and mechanical properties of the recombinant structural protein BP1 and evaluated its biodegradability. Because the thermal degradation occurs above 250 °C and the glass transition temperature is 185 °C, BP1 can be molded into sheets by a manual hot press at 150 °C and 83 MPa. The flexural strength and modulus of BP1 were 115 ± 6 MPa and 7.38 ± 0.03 GPa. These properties are superior to those of commercially available biodegradable polymers. The biodegradability of BP1 was carefully evaluated. BP1 was shown to be efficiently hydrolyzed by some isolated bacterial strains in a dispersed state. Furthermore, it was readily hydrolyzed from the solid state by three isolated proteases. The mineralization was evaluated by the biochemical oxygen demand (BOD)-biodegradation testing with soil inocula. The BOD biodegradability of BP1 was 70.2 ± 6.0 after 33 days.

## Introduction

Many commercially available polymers have some associated environmental problems^1,2^. The increase in the production and subsequent disposal of synthetic polymers causes serious environmental pollution because these polymers do not degrade in the natural environment. Accordingly, researchers have begun to focus on the development of biodegradable polymers as environmentally benign materials that can be degraded by microorganisms into carbon dioxide. Furthermore, the production of synthetic polymers contributes to the depletion of fossil resources. To reduce the usage of fossil resources, researchers have also begun to develop bio-based polymer derived from biomass resources.

Biosynthetic poly((*R*)-3-hydroxyl butyrate) (P3HB), which many microorganisms can use as an energy source, has been developed as a potentially biodegradable polymer^3,4^. Chemosynthetic polyesters^5,6^, i.e. poly(lactic acid) (PLA), polycaprolactone (PCL), poly(butylene succinate) (PBSu), and poly(butylene adipate-*co*-butylene terephthalate) (PBAT) have also been developed as biodegradable polymers since the latter part of the twentieth century, and some of them are manufactured from biomass and used commercially. Natural polymers are also used as biodegradable polymers with or without the modification. For instance, starch modified by glycerol or chemosynthetic polymers, i.e. PCL^7,8^, and cellulose partially esterified with fatty acid^9^ are thermoplastic and biodegradable. The poor mechanical and thermal properties of commercially available biodegradable polymers limit their adoption. Therefore, these biodegradable polymers have only been used as alternatives to general-purpose polymers. PLA, which has a relatively high glass transition temperature (*T*_g_), flexural strength, and flexural modulus i.e. 60 °C, 80–100 MPa, and 3 GPa, is used for applications requiring rigid material^10,11^. However, the biodegradation of PLA is limited to high-temperature compost environments, and PLA does not show biodegradability in the natural environment.

Natural protein materials, such as silk, have been used for fiber material since ancient times. They are bio-based and degrade in the natural environment^12^. Although soybean isolate is an abundant protein resource, it is impossible to use it industrially due to poor processability. Therefore, chemical modification and polymer blending procedures were studied to endow the material with moldability and improved mechanical properties while maintaining biodegradability^13^.

Natural structural proteins, such as elastin, resilin, mussel byssus thread, squid suckerin, silks produced by various insects, and others are gaining attention due to their remarkable mechanical properties^14–19^. For instance, some spider species produce silk fiber with tensile strength of 1.1 GPa and toughness of 160 MJ m^−3^, stronger and tougher than any commercially available fiber^20^. Additionally, biodegradability of naturally occurring structural proteins has been demonstrated in some environments. For example, susceptibility of wool to fungal breakdown^21^ and degradation of regenerated silkworm silk^22^ have been demonstrated. On the other hand, while the performance of natural protein material is high, the low productivity by the aforementioned mammals and insects prevents commercial use. To manufacture structural protein materials, microbial fermentation using genetic engineering has been developed. Genetic engineering allows customization of some properties, i.e. processability, mechanical properties, thermal properties, and hydrophilicity^23,24^ even beyond what is observed in natural proteins. Commercial scale production of protein materials is just starting globally.

Spiber Inc. is developing recombinant structural protein under the trade name Brewed Protein from sugar via a microbial fermentation process^25–28^. The recombinant structural protein material can be spun or molded into fiber, sheet, and bulk material and used as an alternative to conventional plastics. The mechanical properties of protein material are controlled by its structure and amino acid sequence. Protein material is composed of natural amino acids linked together by peptide bonds. Even though amino acids can be metabolized to inorganic compounds by micro-organisms, suggesting that protein material is potentially biodegradable in the natural environment, the cleavage of peptide bonds in a given bulk protein material is not entirely ensured. To employ any novel polymer as a biodegradable polymer, it is necessary to evaluate the degradability of the material in the natural environment.

In this study, we evaluated the thermal and mechanical properties of the recombinant structural protein material BP1 using thermogravimetric analysis (TGA), differential scanning calorimetry (DSC), wide angle X-ray diffraction (WAXD), and three-point flexural testing, and we evaluated the biodegradability of BP1. Hydrolysis by isolated bacteria was evaluated using a clear zone method with BP1-containing emulsified media. The enzymatic degradation was evaluated using compression-molded sheet samples. The aerobic biodegradability of BP1 in an aqueous medium using a soil inoculum was determined by measuring the oxygen demand in a closed respirometer.

## Results and discussion

### Sequence structure

**Figure 1:**
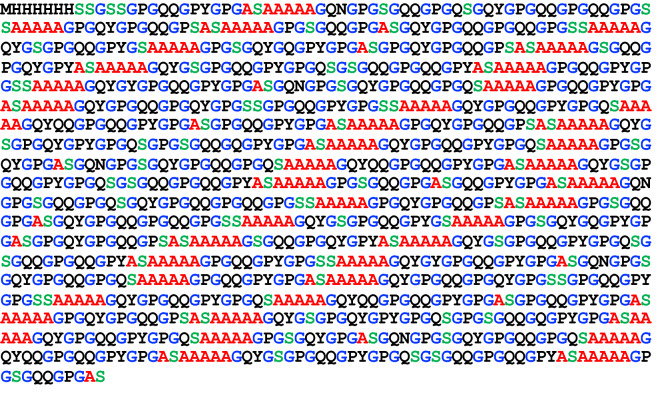
Amino acid sequence of BP1 using single-letter codes.

The sequence structure of BP1 is presented in Fig. 1. BP1 is composed of 1,188 amino acid and the molecular weight of it is 1.06 × 10^5^. The sequence of BP1 is roughly based on natural spider silk. Natural silks include a repetitive central region with alternating blocks of beta-sheet crystallite forming alanine-rich segments and amorphous segments. This repetitive region is flanked by non-repetitive regions on the C and N-termini. In designing BP1, the non-repetitive regions were removed, and the size and amino acid composition of the alanine heavy and amorphous regions have been modified to promote protein expression and reduced-cost purification while maintaining a range of downstream applications.

### Thermal and mechanical properties

**Figure 2:**
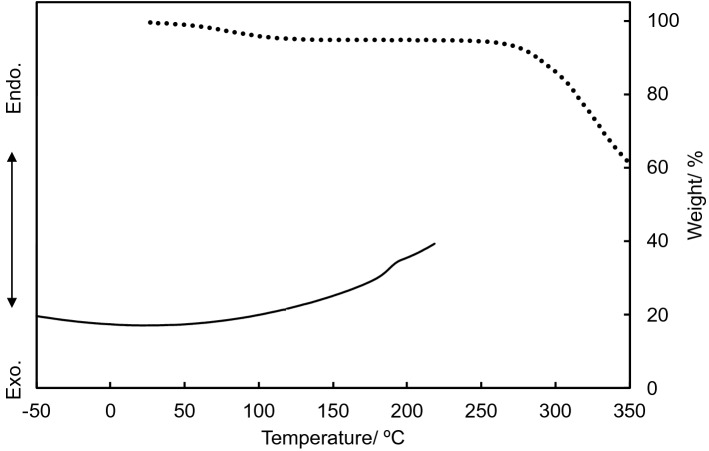
Thermogravimetric analysis (dotted line) and differential scanning calorimetry (solid line) thermograms of BP1.

The thermal and mechanical properties of BP1 are summarized in Table 1. The thermal properties and water content were evaluated by the thermogravimetric analysis (TGA) and differential scanning calorimetry (DSC). Figure 2 shows the TGA curve of BP1 with two steps of weight loss. The initial weight loss below 120 °C corresponds to the evaporation of absorbed moisture. In addition to the amide linkage between amino acids, polar amino acid residues glutamine (23.7%) and serine (9.3%) lead to hydrophilicity and moisture absorption. The second weight loss step above 250 °C is attributed to the degradation of BP1 with 10% weight loss at 285 °C. With evaluation by DSC up to 220 °C, the melting temperature was not observed during the measurement. The glass transition temperature (*T*_g_) was determined to be 185 °C which is far higher than that of the commercially available biodegradable polymers, i.e. that of P3HB, PLA, PBSu, and PCL are 4, 55, − 32, and − 65 °C, respectively^3,5,29–31^, and than that of polyamide as an conventional engineering plastic, i.e. that of nylon-6 and nylon-6,6 are 41 and 48 °C, respectively^32^. Whereas these biodegradable polymers are in the rubbery state at room temperature or have transitions from the glassy to rubbery state just above room temperature and these polyamides soften up under 186 °C, the bulk BP1’s high-temperature transition to the rubber state allows potential use as a conventional engineering plastic like nylon and opens a new potential set of applications for an environmentally biodegradable polymer.

**Table 1:** Thermal and mechanical properties of BP1.

Water content^a, b^/%	10% weight loss temperature^b^/°C	*T*_*g*_^c^/°C	Flexural strength^d^/MPa	Flexural modulus^d^/GPa
6	285	185	115 ± 6	7.38 ± 0.03

^a^Calculated by the weight loss below 120 °C.

^b^Measured by thermal gravity analysis (TGA).

^c^Measured by differential scanning calorimetry (DSC).

^d^Measured by three-point flexural strength testing.

**Figure 3:**
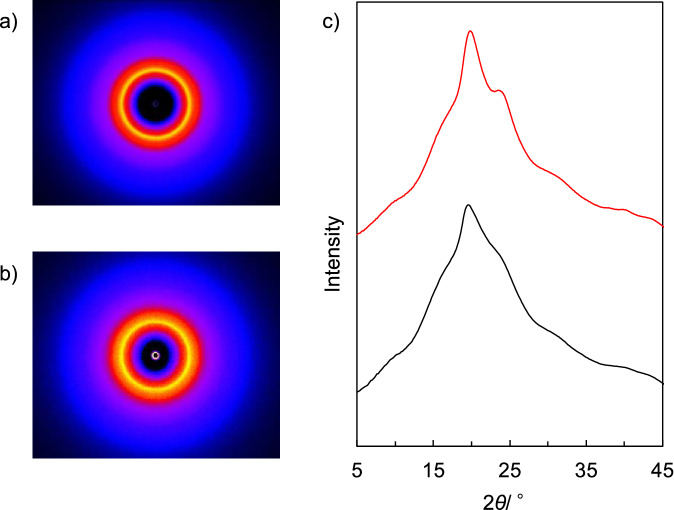
2D WAXD images of (**a**) compression-molded BP1 (**b**) BP1 powder. (**c**) 1D WAXD patterns of the compression-molded BP1 (red line) and the BP1 powder (black line).

As shown in Fig. 1, BP1 is a block copolymer of an alanine-heavy region capable of forming beta-sheet crystallites and an amorphous region, yielding a semi-crystalline polymer. However, the endothermic peak derived from the melting point was not observed in DSC analysis. Figure 3 shows the WAXD patterns of the compression-molded BP1 sheet used for enzymatic biodegradation testing and for biochemical oxygen demand (BOD) biodegradation testing and BP1 powder. The crystallinity of the compression-molded BP1 sheet and the BP1 powder was calculated from WAXD measurement of 5 samples resulting in 29.3 ± 1.1% and 20 ± 1.3%, respectively. These results indicate that BP1 samples used for the evaluation of biodegradability are semi-crystalline polymer and that the melting temperature is above the decomposition temperature.

The mechanical properties of BP1 were evaluated by three-point flexural strength testing. The strength and modulus of the BP1 sheets were 115 MPa ± 6 MPa and 7.38 ± 0.03 GPa, respectively. When compared to other bio-based materials, such as PLA having flexural strength of 80–100 MPa and flexural modulus of 3 GPa^10,11^ and PHB with strength of 60 MPa and modulus of 5.3 GPa^33^, BP1 shows both higher strength and modulus. The mechanical properties of the BP1 sheets are superior to PLA, which has higher thermal properties than other commercially available material, and P3HB, which has good biodegradability in the natural environment. Overall, with thermal stability beyond 180 °C and 10% weight loss at 285 °C, of BP1 has a potentially wide temperature range of applications including as a conventional engineering plastic.

### Hydrolyzability of emulsified BP1 by bacterial strains

Like other biodegradable polymers, proteins are degraded in the natural environments via the following 2 steps. In the first step, they are degraded by hydrolytic enzymes to give shorter and hydrolyzed protein chains. The hydrolyzability of BP1 was evaluated by the clear zone method using emulsified medium containing BP1 streaked with 5 bacterial strains. *S. griseus* generates Pronase E, which non-specifically degrades proteins, including *Bombyx mori* silk materials^34,35^. The size of the resulting clear zones after incubation for 24 h with various bacterial strains is summarized in Table 2. Strains NBRC 13287, NBRC 100445, NBRC 100991, and NBRC 100993 are recommended by ASTM protocol for degradation testing^36^. Strains NBRC 100445 and NBRC 100993 formed large clear zones and strains NBRC 13287, NBCR 12875 formed medium clear zones after incubation for 24 h, while strain NBRC 100991 did not form a clear zone. These results indicate that BP1 in an emulsified state is susceptible to be degraded by some environmental bacteria.

**Table 2:** Clear zone formation ability on the BP1-containing media after 24 h.

Bacteria strains	Species	Size of clear zone/mm
NBRC 13287	*Vibrio proteolyticus*	+
NBRC 12875	*Streptomyces griseus*	+
NBRC 100445	*Bacillus* sp.	++
NBRC 100991	*Pseudomonas* sp.	–
NBRC 100993	*Pseudoalteromonas haloplanktis*	++

++, large clear zone (> 4.0 mm) formed; +,  medium clear zone formed (3.0–4.0 mm); –, no clear zone.

### Enzymatic degradability of compression-molded BP1 sheets

**Figure 4:**
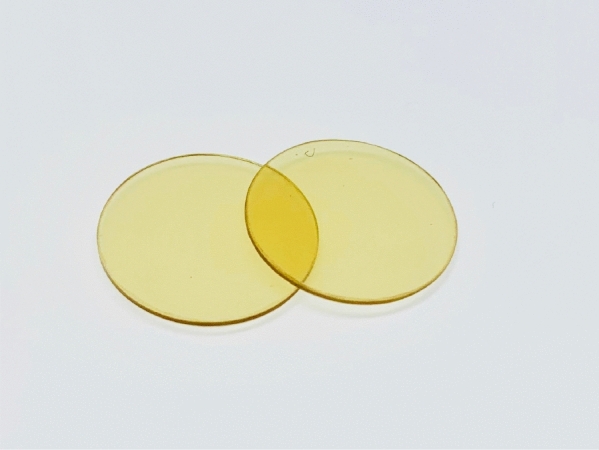
Photograph of compression-molded BP1 sheets used in enzymatic degradation testing.

Although the clear-zone method with polymer-emulsion-containing plates is a powerful tool for the evaluation of biodegradation by extracellular enzymes produced by isolated bacteria, it is not sufficient for biodegradability assessment. Some enzymes are capable of attacking biodegradable polymer in an emulsified state, but not in the solid state, i.e., Alejandra et al. reported the enzymatic degradation of P3HB by commercial lipase^37^, but Mukai et al. reported most commercial lipases could not degrade P3HB sheet^38^. Therefore, we evaluated the enzymatic degradability of the melt-pressing sheet as a solid-state sample using three enzymes. The compression-molded BP1 sheets are shown in Fig. 4. Proteinase K, which is a serine protease, can degrade the structural protein keratin and biodegradable poly(*L*-lactic acid) PLLA. Pronase E and chymotrypsin are also serine proteases and degrade silk fibroin^39^.

**Figure 5:**
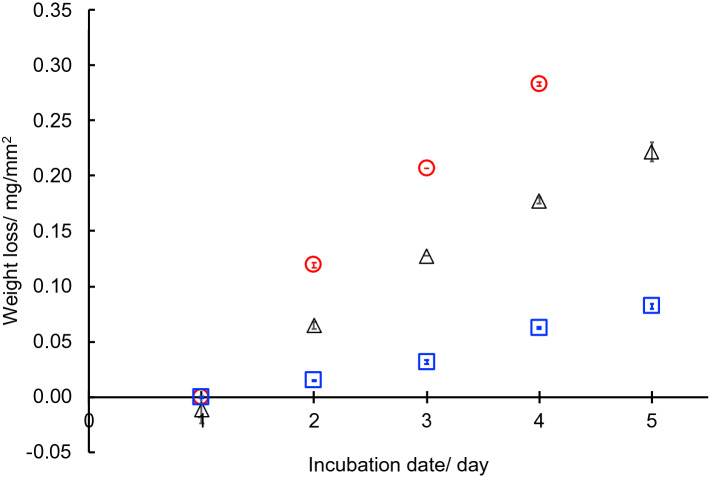
Enzymatic degradation of the BP1 sheets with Proteinase K (red circle), Pronase E (black triangle), and chymotrypsin (blue square) at 37 °C. Error bars indicate standard error. All experiments were performed in triplicate (n = 3).

The weight loss of the BP1 sheets after incubation for 5 days was shown in Fig. 5. The BP1 sheets were hydrolyzed by all three enzymes. In the case of Proteinase K, the sheets could not be recovered on the fifth day because of hydrolysis and fragility. The rate of weight loss with Proteinase K, Pronase E and chymotrypsin were 0.066 mg mm^−2^ day^−1^, 0.042 mg mm^−2^ day^−1^, and 0.014 mg mm^−2^ day^−1^ versus 0.000 mg mm^−2^ day^−1^ loss for control samples immersed in buffer without any enzyme. Li et al. reported that the rate of weight loss of PLLA with Proteinase K was 0.007 – 0.053 mg mm^−2^ day^−1^^40^. These results show that semi-crystalline and solid-state BP1 sheets can be hydrolyzed by some proteinases. Similar to a previous report on *Bombyx mori* silk by Guo et al.^41^, it was observed that Proteinase K is more efficient in degrading BP1 as compared to chymotrypsin. These results suggest that BP1 can degrade in the conditions where PLLA degrades, like compost.

### BOD biodegradation testing of BP1 with fresh water

The clear zone formation method by isolated bacteria and the enzymatic sheet degradation testing indicate that BP1 can be enzymatically hydrolyzed to shorter protein chains in the emulsified and solid state, respectively. Although amino acids and oligopeptides are generally metabolized by various microorganisms in any natural environment^42^, mineralization should be confirmed in order to claim the target polymer is biodegradable. The BOD method based on ISO 14851 was used to assess the BOD biodegradability of both BP1 and a mixture of free amino acids of BP1 composition.

**Figure 6:**
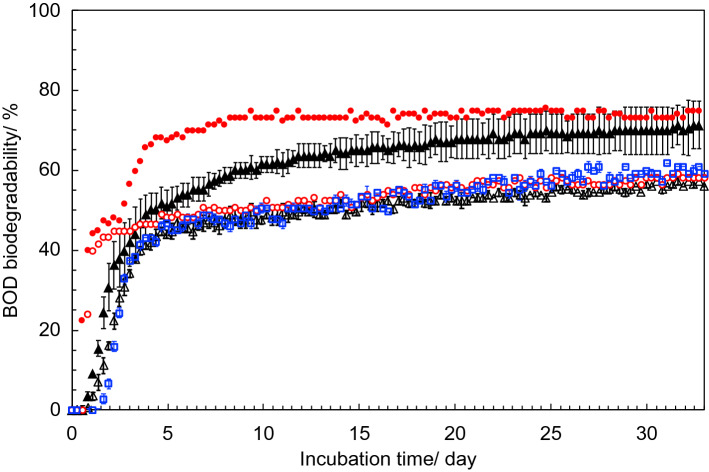
BOD biodegradation curves of amino acids with mixed soil and NH_4_Cl, 5.0 mg L^−1^ (open circle), amino acids with single-source soil and NH4Cl, 0.50 mg L^−1^ (closed circle), BP1 with mixed soil and NH4Cl, 5.0 mg L^−1^ (open square), BP1 with single-source soil and NH_4_Cl, 5.0 mg L^−1^ (open triangle), and BP1 with single-source soil and NH_4_Cl, 0.50 mg L^−1^ (closed triangle) at 25 °C for 30 days. Error bars indicate standard error.

The BOD biodegradation curves are shown in Fig. 6, and the final degree of biodegradation is summarized in Table 3 including measured data and data corrected for interference by nitrification. Ammonium released by biodegradation of the nitrogen-containing protein material can undergo nitrification and be further oxidized to NO_2_^−^ and then NO_3_^−^ by the following reactions,

1 $${\text{2 NH}}_{{4}} {\text{Cl }} + {\text{ 3 O}}_{{2}} = {\text{ 2 HNO}}_{{2}} + {\text{ 2 HCl }} + {\text{ 2 H}}_{{2}} {\text{O}}$$

2 $${\text{2 HNO}}_{{2}} + {\text{ O}}_{{2}} = {\text{ 2 HNO}}_{{3}}$$

**Table 3:** BOD biodegradability of the mixture of amino acids and BP1 with and without nitrification correction.

	Mixture of amino acids/%		BP1/%		
NH_4_^+^/mg L^−1^	5.0	0.50	5.0	5.0	0.50
Sampling points of Inocula	Kiryu Hamamatsu Wakayama	Kiryu	Kiryu Hamamatsu Wakayama	Kiryu	Kiryu
Incubation period/day	40	33	40	49	33
Without nitrification correction	60.6^b^	74.7	57.9 ± 0.0^c^	64.6 ± 1.0^d^	71.2 ± 5.9
With nitrification correction^a^	59.4	73.7	56.8 ± 0.1	63.4 ± 1.0	70.2 ± 6.0

^a^corrected by ion chromatography using the resulting solution after each period. ^b^58.1 % at 33 days. ^c^57.2 ± 0.0 % at 33 days. ^d^60.8 ± 0.0 % at 33 days.

The oxygen consumed by these reactions is included in the total biochemical oxygen demand, and thus ISO 14,851 requires quantification of NO_2_^−^ and NO_3_^−^ concentration in the final test liquid. The final oxygen demand should be modified to remove BOD due to nitrification reactions before calculation of degree of biodegradation.

The BOD biodegradation of the mixture of amino acids started after 0.5 days and reached a plateau phase after 1.5 days. That of BP1 started after 1 day and reached a plateau phase after 4 days. Amino acids as a major source of nutrients can be smoothly assimilated and metabolized by bacteria, resulting in its biodegradation within short incubation times. As for the protein material, because the biodegradation of polymer involves two steps, the biodegradation started relatively later and required more time compared to that of amino acids. The BOD biodegradability of the mixture of amino acids and the protein material using three-site inocula after incubation for 40 days at 25 °C reached 60.6 and 57.9 ± 0.0% and that modified by nitrification was 59.4 and 56.8 ± 0.1%, respectively. These results suggest that the metabolic turnover from hydrolysates that were generated by hydrolysis of BP1 to inorganic compounds are the same as those of the mixture of corresponding amino acids. The BOD biodegradability using single-site inoculum of BP1 was 64.6 ± 1.0% and that modified by nitrification was 63.4 ± 1.0%. These results indicate that the biodegradability of BP1 does not depend on the degradation environment.

Although the BOD biodegradability of BP1 reached around 60% after 40 days, this is short of the criteria for a chemical as defined by the OECD 301 test methods^43^. Especially the biodegradability of the mixture of amino acids, which is easily metabolized by bacteria, should be higher than 60%. Since the ammonium chloride affects the metabolism of bacteria, we reduced the concentration of NH_4_Cl for the BOD biodegradation testing to one-tenth the concentration defined in ISO 14851.

The BOD biodegradation curves with low concentration of NH_4_Cl are also shown in Fig. 6, and the final BOD biodegradability and the modified BOD biodegradability are summarized in Table 3. The biodegradation of the mixture of amino acids and BP1 started after 0.5 days and reached a plateau phase after 4 days. The modified BOD biodegradability of the mixture of amino acids and BP1 after 33 days were 73.7 and 70.2 ± 6.0%, respectively. Although the effect of ammonium salt for biodegradability is not mentioned in ISO 14851 and other ISO protocol, the concentration of ammonium salt had an effect on the BOD biodegradability of the nitrogen containing BP1. In order to obtain the correct biodegradability value, reduction of the NH_4_Cl concentration in the test medium should be considered.

Moreover, a systematic study of the relationship between ammonium concentration in test medium and degree of biodegradability of protein is called for.

## Conclusion

Bio-based and biodegradable materials are essential to establish a circular economy in pursuit of a more sustainable society. The recombinant structural protein (BP1) tested in this study is a bio-based material, as it is manufactured via a microbial fermentation process with glucose as the feedstock. The mechanical properties of currently available biodegradable materials are suitable for use as general-purpose polymers. Because the glass transition temperatures of commercially available biodegradable plastics except for PLA are below room temperature, they are pliable at room temperature and thus used as alternatives of polyethylene. Although the glass transition temperature of PLA is 60 °C and it is relatively rigid at ambient temperatures, it cannot degrade in the natural environment—only in compost at high temperature.

On the other hand, BP1 can be molded into sheets with 115 MPa flexural strength and 7.38 GPa flexural modulus. BP1 has thermal stability up to 250 °C and 10% weight loss at 285 °C, having a potentially wide temperature range of applications including as a conventional engineering plastic. Several isolated bacteria and enzymes hydrolyze BP1, indicating that it can be quickly biodegraded to amino acids and oligopeptides. The BOD biodegradability of BP1 is up to 70.2 ± 6.0% with nitrification correction.

The BP1 sheet is a thermally stable material with mechanical properties greater than those of commercially available biodegradable plastics and with good biodegradability in the natural environment, indicating that BP1 is a promising bio-based and biodegradable material.

## Methods

### Reagents and materials

BP1 was produced by a fermentation process using *E.coli*, which was previously reported in the literature^28,44^ and provided in an unprocessed powder form by Spiber Inc. The amino acid composition of BP1 was alanine (16.4%), tyrosine (12.0%), glutamine (23.7%), glycine (21.9%), proline (15.3%), serine (9.3%) and others (1.4%). The protein sequence structure is shown in Fig. 1.

KH_2_PO_4_, K_2_HPO_4_, NaCl, Na_2_HPO_4_·H_2_O, NH_4_Cl, MgCl_2_·6H_2_O, CaCl_2_, FeCl_3_·6H_2_O, Yeast extract, and agar powder were purchased from FUJIFILM Wako Pure Chemical Co. (Osaka, Japan). Na_2_SO_4_, KCl, HCl, Na_2_SO_4_, NaHCO_3_, and NaOH were purchased from Kanto Chemical Co., Inc. (Tokyo, Japan). Plysurf was purchased from DKS Co. Ltd. (Kyoto, Japan). All chemicals were of reagent grade and used without further purification. Pronase E (P5147 Protease Type XIV from *Streptomyces griseus*) was purchased from Sigma Aldrich, Proteinase K was purchased from TaKaRa Bio Inc (Kusatsu, Japan), and chymotrypsin was purchased from NACALAI TESQUE, INC (Kyoto, Japan).

### Thermal analysis

The thermal stability was determined using a thermogravimetric analyzer (DT-60H Thermogravimetry Analyzer; Shimadzu Co., Ltd., Kyoto, Japan), whereby the powder sample (8.0 mg) without additional drying process was heated to 350 °C under compressed air flow in an open aluminum pan at a rate of 20 °C min^−1^. Double the mass of Al_2_O_3_ was used as the reference sample. The water content was calculated from the weight loss below 120 °C. The glass-transition temperature (*T*_g_) was determined by differential scanning calorimetry (DSC-8500; PerkinElmer Japan Co., Ltd., Yokohama, Japan). The powder sample (5.9 mg) was dried in a vacuum oven for 2 h and was heated to 220 °C under N_2_ at a rate of 20 °C min^−1^.

### Crystallinity measurement

The WAXD measurements were conducted using an X-ray diffractometer (Rigaku XtaLAB-SP X-ray) to determine the degree of crystallinity (*X*_c_) using Cu-Kα radiation with a wavelength of 0.154 nm and exposure time of 100 s. The voltage was set to 40 kV and the current 30 mA. BP1 powder or milled thin sheet was filled into the well of the sample substrate and scanned from 5° to 45°. The crystallinity (*X*_c_) of BP1 was estimated after the deconvolution by Gaussian–Lorentzian mixed function in accordance with the following equation:

3 $$X \, \left( \% \right) = \, \left[ {A_{{\text{c}}} /\left( {A_{{\text{c}}} + A_{{\text{a}}} } \right)} \right] \, \times { 1}00$$

where *A*_c_ and *A*_a_ are the total area of diffractions for the amorphous region and the crystalline region, respectively.

### Mechanical properties

Flexural testing samples were prepared and tested in accordance with ISO 178. The specimens (250 × 350 × 1.75 mm) were molded into sheets by a manual hot press at 150 °C and 83 MPa. The samples were stored at 20 °C and 60% RH for 48 h. A three-point bending test was performed by using a universal testing machine (AUTOGRAPH AGS-X; Shimadzu Co. Kyoto, Japan) with the measurement rate of 1.0 mm/min at 20 °C.

### Preparation of protein-emulsified media

BP1 (1.0 g) was dissolved in dichloromethane (100 mL). The solution was emulsified with an ultrasonic disruptor (UD-200; TOMY Seiko Co. Ltd., Tokyo, Japan) in a basal medium (1.00 L) at pH 7.0 with the following components: Na_2_HPO_4_·12H_2_O, 11.6 g L^−1^; KH_2_PO_4_, 4.6 g L^−1^; MgCl_2_·7H_2_O, 0.50 g L^−1^; NH_4_Cl, 1.0 g L^−1^; FeCl_3_·6H_2_O, 0.10 g L^−1^; Yeast extract, 0.50 g L^−1^; and 1% Plysurf, 5.0 mL L^−1^. Dichloromethane was removed by heating at room temperature with a magnetic stirrer. Agar (1.50 g) was added to the emulsified medium, and the mixture was autoclaved at 121 °C for 15 min. The BP1 media was solidified in a petri dish after cooling.

### Hydrolysis of BP1 by different bacterial strains

Five bacterial strains were acquired from the Biological Resource Center, NITE (NBRC) strain collection in lyophilized form (NBRC 13287, NBRC 12875, NBRC 100445, NBRC 100991, and NBRC 100993) and were resuspended according to supplier’s instructions. Strains were streaked onto the plates using wooden stick, and plates were incubated at 30 °C. The ability of each strain to hydrolyze BP1 was assessed by measuring the size of the clear zone formed after 24 h.

### Enzymatic degradation of BP1 sheet

BP1 was molded into thin sheets by compression molding at 150 °C and 83 MPa. and cut to square samples with a size of 1.0 × 1.0 cm × 0.5 mm. Pronase E, Proteinase K, and chymotrypsin were used for the sheet degradation testing. The buffer solution was prepared with the following components: NaCl, 8.0 g L^−1^; KCl, 0.2 g L^−1^), Na_2_HPO_4_, 3.58 g L^−1^; KH_2_PO_4_, 0.24 g L^−1^, and the pH was adjusted using HCl to 7.4. The enzymes were diluted in Milli-Q water to prepare the stock solution of the enzymes (20 g L^−1^), and the stock solution (7.5 μL) was diluted with the buffer solution to 1.0 mL. The sample sheets (1 × 1 cm) were put in the buffer solution (1.0 mL) and incubated at 37 °C. The incubated sheets were collected every 24 h to weigh the sheets and to replace the enzyme solution and average mass loss of three sheets per each sample was recorded.

### BOD-biodegradation testing with soil inoculum

The biochemical oxygen demand (BOD) biodegradability by aerobic microorganisms from soil suspension in an aqueous medium was determined by measuring oxygen consumption with BOD instrumentation (OxiTop-C measuring head with a 300-mL BOD reactor, WTW GmbH, Weilheim, Germany) referring to ISO 14851 standard. Thin protein sheets produced by the method described above were prepared for testing by cryogenic milling in accordance with ISO 10210:2012. The resulting particles were sieved to get particle size below 250 μm in accordance with ISO guidelines. The following stock solutions were prepared: solution A1; KH_2_PO_4_, 8.5; K_2_HPO_4_, 21.75; Na_2_HPO_4_·H_2_O, 33.4; NH_4_Cl, 0.5 g L^−1^, solution A2; KH_2_PO_4_, 8.5; K_2_HPO_4_, 21.75; Na_2_HPO_4_·H_2_O, 33.4; NH_4_Cl, 0.05 g L^−1^, solution B; MgSO_4_·7H_2_O 22.5 g L^−1^, solution C; CaCl_2_, 27.5 g L^−1^, solution D; FeCl_3_·6H_2_O, 0.25 g L^−1^. To adequate amounts of deionized water, 2 mL of solution A1 or A2 and 0.2 mL each of solution B, C, and D were added, and the solution was made up to 200 mL with water. A sample (ca. 6.5 mg) was placed in the 300-mL BOD reactor, and 200 mL of BOD medium was added to the reactor. To prepare the inoculum for BOD biodegradation testing, soil (1.0 g) from Kiryu City (“single-source soil”) or a mixture of soil (1.0 g) from Kiryu City, Hamamatsu City, and Wakayama City, called “mixed soil”, was dispersed in distilled water (10 mL) and filtered through a filter paper. Then, the filtrate was allowed to stand for 18 h. The supernatant of the soil solution (200 μL) was added to a BOD reactor as an inoculum, OxiTop-C measuring heads were attached on the heads of a BOD reactors, and the reactor was incubated at 25 °C. The concentrations of NO_3_^−^ and NO_2_^−^ of the resulting solution were measured by ion-chromatography. As a control sample, a mixture of free amino acids was used in proportion to their weight percent in the protein sequence structure. The control sample was tested as N = 1 and BP1 sample was tested as N = 2. Those listed as ‘others’ in Reagents and materials section were neglected.
